# Special Issue “Genetic Advances in Neuromuscular Disorders: From Gene Identification to Gene Therapy”

**DOI:** 10.3390/genes12020242

**Published:** 2021-02-08

**Authors:** Virginia Arechavala-Gomeza, Lidia Gonzalez-Quereda

**Affiliations:** 1 Neuromuscular Disorders Group, Biocruces Bizkaia Health Research Institute, 48903 Barakaldo, Spain; 2 Ikerbasque, Basque Foundation for Science, 48009 Bilbao, Spain; 3 CIBERER, Universitat Autónoma de Barcelona (UAB) IIB Sant Pau, Hospital de Sant Pau, 08041 Barcelona, Spain; lgonzalezq@santpau.cat

Since the gene responsible for Duchenne muscular dystrophy was first described in 1987 [1], knowledge of neuromuscular diseases (NMD) has evolved rapidly. Advances in molecular genetics and an ongoing revolution in diagnostic techniques have facilitated the identification of a large number of causative genes in recent years and treatments are being developed against many new targets. However, the large number of genes related to these diseases, some of them of great size and complexity, as well as the high clinical and genetic heterogeneity that these entities usually present, pose a challenge for clinicians and researchers when defining all the intricacies that make up these diseases.

This Special Issue “Genetic Advances in Neuromuscular Disorders: From Gene Identification to Gene Therapy” includes 15 high-quality papers that seek to deepen the knowledge of multiple aspects related to some of the most prevalent and disabling neuromuscular diseases.

At a time when the development of gene therapy is booming and various mutation-dependent therapies are becoming available [2], establishing a detailed natural history of these entities, determining a specific genetic cause, and understanding molecular defects are of utmost importance. In this regard, the work of Knyazeva et al. extends the knowledge of the role of Filamin C (FLNC) in muscle cells, focusing on muscle alterations developed after FLNC loss. The authors confirm the role of FLNC in the regulation of essential muscle processes such as muscle cell proliferation, migration, and differentiation [3]. Jorholt et al. contribute to the clinical knowledge of ALPK3-associated cases by reporting two new cases of hypertrophic cardiomyopathy with muscle involvement and variability in age of onset [4]. Knowing the factors involved in the pathogenesis and progression of these diseases is paramount for their prevention, management, and treatment. Interestingly, the work of Ignatieva et al. demonstrates how LMNA mutations associated with different clinical phenotypes affect the molecular mechanisms involved in skeletal muscle differentiation and metabolism [5]. With the aim of achieving a rapid and cost-effective genetic diagnosis, the work of Alcántara-Ortigoza et al. proposes an combination of multiplex ligation-dependent probe amplification (MLPA) and Next-Generation Sequencing (NSG) as a first-line diagnostic approach for Mexican patients with muscular dystrophy, displacing muscle biopsy to a second tier of the diagnostic process [6]. Taking advantage of new technologies also, Gonzalez-Quereda et al. analyze the mutational spectrum of a cohort of 207 patients suffering from NMD by evaluating a custom targeted resequencing neuromuscular gene panel as a diagnostic tool. They established a conclusive genetic diagnosis in about 50% of patients, with RYR1 and TTN accounting for almost 30% of cases [7]. Mroczek et al. review the particular molecular structure of PLEC, as well as its tissue-specific functions and associated phenotypes. The authors also investigate the role of plectin deficiency in congenital myasthenic syndromes by describing four patients carrying the PLEC c.1_9 mutation in homozygosity and suggesting a common origin of the mutation [8]. García-Solaesa et al. describe, for the first time in a Spanish population, a new splice variant associated with a myopathic form of PGK1 deficiency. The authors also provide an interesting discussion on the dichotomization of PGK1 into myopathic forms without anemia and hemolytic forms [9].

One of the challenges of medicine is to identify physiological or pathological processes through the use of biomarkers that are specific, sensitive, and predictive. The work of Pegoraro and Angelini determines the role of miR206 in muscle recovery and regeneration, pointing to it as an easily accessible circulating biomarker for prognosis to monitor the progression of muscle damage in limb-girdle muscular dystrophies [10]. With regard to one of the most frequent and disabling muscular dystrophies of childhood, Duchenne muscular dystrophy, several studies allow us to deepen our knowledge of various aspects of the disease. Lim. et al. review the literature on cardiac involvement in DMD symptomatic women and discuss the implications of studies of female DMD carriers in the development of therapies aimed at increasing cardiac dystrophin levels [11]. The work of Chengmei et al. reviews the current status of DMD pathogenesis and therapy, focusing on the mutational spectrum; diagnostic tools, clinical trials, and therapeutic approaches; and also evaluating the clinical potential of new advanced therapeutic strategies [12]. Lim. et al. review the animal models developed for DMD that have been created using CRISPR-based technology, a topic of special interest, given that having reliable animal models is key to exploring new therapeutic targets [13]. 

DM1 is one of the most common adult forms of muscular dystrophy and the expansion of a specific trinucleotide sequence (CTG) is the molecular pathological mechanism responsible for its clinical manifestations. Expansions can be large and complex and, even today, their correct characterization in size and composition is a diagnostic challenge. On this topic, Ballester-Lopez et al. compare five different approaches to estimate CTG expansion size in 15 patients with DM1. They found variability between the methods used and, based on their results, propose small pool (SP)-PCR as the most appropriate technique [14]. Castro et al. review advances in the understanding of the pathogenic mechanisms associated with repeat expansion neurodegenerative and neuromuscular diseases, focusing on the impact of antisense repeat transcription in the development of new therapies [15]. López-Martinez et al. review the role of tissue-specific regulators of alternative splicing of MBNL and CELF1, and describe the molecular mechanisms underlying the misregulation of this process. In addition, they provide a list of transcriptional alterations implicated in the pathogenesis of DM1 [16]. The work of Ballester-Lopez et al. studies the variability of CTG expansion in three different tissues (blood, muscle, and skin) from eight patients with DM1 and its association with the patient’s clinical characteristics. They suggest that progenitor CTG size, measured in muscle tissue, is associated with age at disease onset and functional impairment of muscle [17]. 

In summary, this Special Issue delves into some of the most relevant aspects concerning neuromuscular diseases today, offering an overview of the current landscape and shedding light on the path from gene identification to gene therapy.
